# Myxedema Coma: Case Report and Literature Review

**DOI:** 10.7759/cureus.15277

**Published:** 2021-05-27

**Authors:** Sherif Elkattawy, Preanka Dhanoa, Juliet Kotys, Hardik Fichadiya, Ari Eckman

**Affiliations:** 1 Internal Medicine, Rutgers-New Jersey Medical School/Trinitas Regional Medical Center, Elizabeth, USA; 2 Internal Medicine, St. George's University, True Blue, GRD; 3 Internal Medicine, Trinitas Regional Medical Center, Elizabeth, USA

**Keywords:** myxedema coma, hypothyroidism, congenital hypothyroidism, thyroid-stimulating hormone (tsh), free t4

## Abstract

Myxedema coma is a life-threatening manifestation of hypothyroidism associated with altered mental status, hypothermia, and symptoms related to the slowing of other organ systems. It can occur as a culmination of severe, longstanding hypothyroidism or be precipitated by acute stressors such as infection, myocardial infarction, cold exposure, and surgery in patients with poorly controlled hypothyroidism. Given the high mortality rate and acuity with which the disease presents, treatment with thyroid hormone replacement should be initiated upon suspicion of the disease even prior to obtaining laboratory confirmation. Stress doses of hydrocortisone should also be given until coexisting adrenal insufficiency is excluded. We present a case of a 58-year-old male who presented to the emergency department after being found on the floor of his house. Physical examinations and laboratory results were significant for myxedema coma and the patient was given levothyroxine with improvement of symptoms and mild change in thyroid hormone levels during hospitalization.

## Introduction

Hypothyroidism is a condition involving low thyroid hormone levels which can result in dysfunctions in heart rate, body temperature, and metabolism. The most common causes of hypothyroidism in the United States and worldwide are autoimmune thyroiditis and iodine deficiency, respectively [1-2]. Other possible causes are medication-induced, pituitary dysfunction, iatrogenic, infectious, postpartum, infiltrative, or congenital. Congenital hypothyroidism is rare due to universal newborn screening and early intervention with levothyroxine. If untreated, the condition presents with intellectual disability, abnormal facies, stunted growth, and abnormal bone growth [1]. Myxedema coma is a life-threatening condition resulting from severe deficiency of thyroid hormone due to chronic undiagnosed or untreated hypothyroidism. It may also be precipitated by infection, trauma, or surgery. A diagnosis of myxedema coma should be suspected in a patient with coma or altered mental status who is also hypothermic, hyponatremic, and/or hypercapnia [2]. However, most patients do not present with myxedema or coma and so the important feature is altered mental status [3]. Due to the life-threatening severity of myxedema coma, treatment with thyroid hormone should begin while awaiting laboratory confirmation. The patient should be admitted to the ICU with intensive pulmonary and cardiovascular support.

## Case presentation

**Figure 1:**
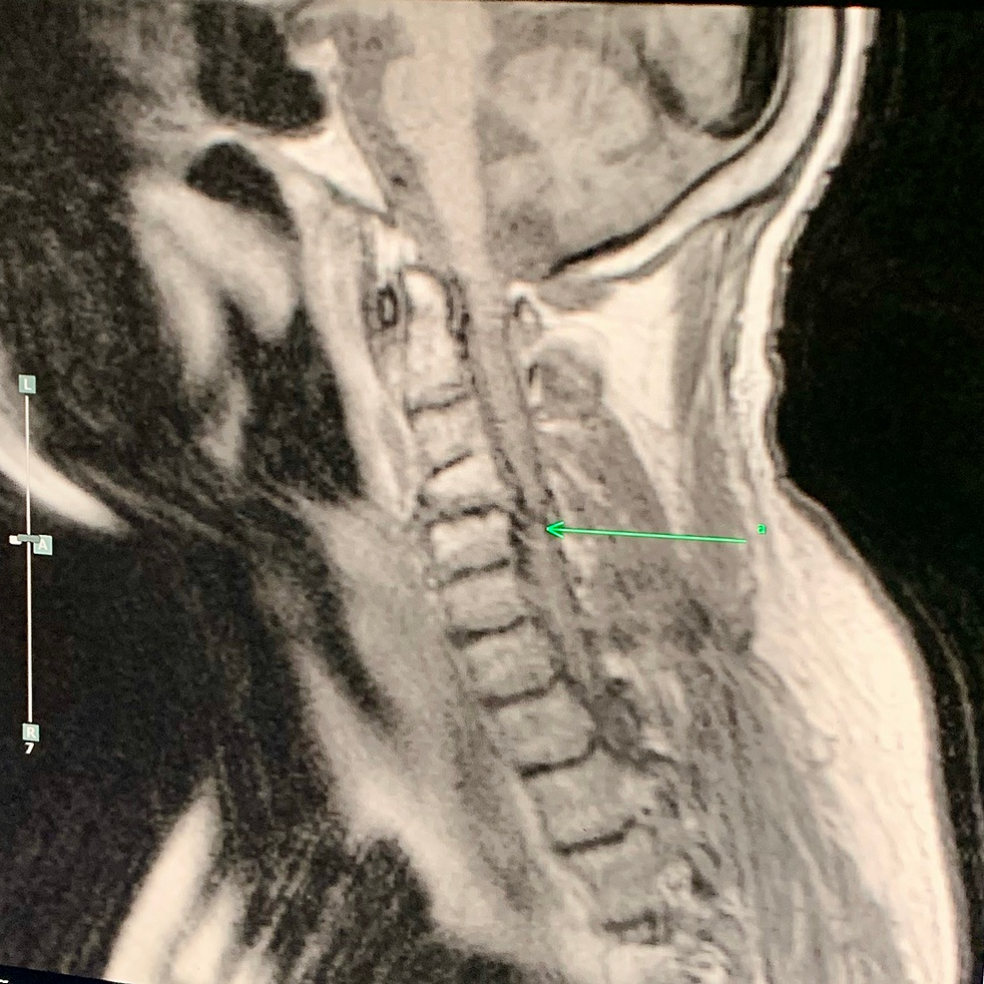
MRI of the cervical spine showed large disc herniation with congenital cervical canal stenosis

A 58-year-old male with a history of untreated hypothyroidism presented to the emergency department after being found on the floor of his apartment confused. The patient was last seen two days prior. In the emergency department, the patient's vital signs were remarkable for a body temperature of 82°F. After receiving a warming blanket and warming fluids, his temperature improved to 89°F. The patient was also bradycardic with stable blood pressure. On examination, the patient was not alert or oriented to person, place, or time, however, he did respond to painful stimuli. Physical examination was remarkable for macroglossia, abnormal facies, short stature, and questionable intellectual disability. Laboratory findings were remarkable for creatinine phosphokinase of 20,000 units/L (55-170 units/L), creatinine of 1.52 mg/dl (0.7-1.2 mg/dL), thyroid-stimulating hormone (TSH) of 97.62 mIU/L (0.4-4 mIU/L) and free T4 of 0.00 ng/dL (0.9-2.3 ng/dL).The patient received hydration, dexamethasone, and a loading dose of 100 mcg IV of levothyroxine, followed by levothyroxine 50 mcg daily. On imaging, MRI of the cervical spine showed large disc herniation with congenital cervical canal stenosis for which the patient was stabilized with a C-spine collar as seen in Figure 1. A CT scan of the abdomen was questionable for ileus for which the patient received nasogastric (NG) tube decompression. The patient was admitted to the ICU for close monitoring and the following morning, he was transferred to the medical floors given the significant improvement of his mental status.

After gradual yet subtle improvements in T4 and TSH over the course of seven days, the patient became hypothermic - rectal temperature was 85.4°F, blood pressure was 83/50 mmHg, and the patient’s pulse rate was 42 beats per minute. The patient was given warming blankets and labs and blood; urine cultures were sent for analysis. The patient was given a bolus of normal saline but remained hypotensive and hypothermic. Adrenal insufficiency workup including cosyntropin stimulation test was within normal limits. Atropine and cosyntropin 0.25 mg IV push were given. The patient was not intubated but was transferred to ICU for closer monitoring and stabilization. The endocrinologist increased the daily levothyroxine dose to 100 mcg daily. The following morning in the ICU, the patient was awake and alert and was transferred back to the medical floors. Blood and urine cultures showed no growth. The patient remained comfortable, alert, and oriented with no complaints. He was later discharged to subacute rehabilitation on oral 125 mcg of levothyroxine daily and outpatient follow up with endocrinology.

## Discussion

The term myxedema coma is a misnomer as neither coma nor myxedema is required for diagnosis. There are no clear diagnostic criteria for the condition, however, patients typically present with altered mental status, confusion, and lethargy [4]. A retrospective study in Japan found the in-hospital mortality rate of myxedema coma to be 30%, which has declined from years prior due to prompt diagnosis and treatment [5]. There is a universal consensus that thyroid hormone replenishment is integral for the treatment of myxedema coma; however, the debate lies in whether to use T4 alone or in combination with T3. There have been reports involving animal studies suggesting adequate tissue perfusion of T3 cannot be obtained in thyroidectomized rats after T4 replacement alone [2]. However, human studies demonstrate that even in the absence of T3, T4 is capable of exerting the required biologic effects for recovery in myxedema patients [2]. T4 has a slow onset of action with few adverse effects, in contrast to T3 which has an affinity for the nuclear receptor that is 10-20-fold higher than that of T4 [6]. 

Although it is indisputable that T3 has superior potency in improving thyroid hormone status, there are risks that must be considered prior to administration. One of the ways in which T3 increases metabolic rate is via increasing oxygen consumption by tissues. This poses an issue if the patient is unable to elicit a compensatory increase in cardiac output in order to meet these increased demands. A mismatch in consumption and delivery of oxygen may precipitate tissue hypoxia and ultimately result in mortality. This is an important consideration for elderly patients and those with cardiac or respiratory comorbidities [7]. In all patients, over-vigorous attempts to reverse hypothyroid state can result in life-threatening complications, therefore, when T3 is indicated, it should be used at a low and gradual dose. As a result of the lack of objective criteria for diagnosis of myxedema coma, response to treatment should be evaluated based on clinical judgment. An appropriate response to treatment would be evidenced by improvement in mental status and stabilization of vital signs. The monitoring of T4 and TSH is an objective way to observe improvement in patients with myxedema coma. However, TSH levels are reflective of the thyroid status for the prior 6-8 weeks, therefore these levels may not be representative of response to thyroid treatment. For this reason, clinical improvement and T4 status may be superior in assessing response to therapy.

Our patient had a long-term history of untreated hypothyroidism with intellectual disability, short stature, abnormal facies, and congenital cervical stenosis which raised suspicion for possible congenital hypothyroidism. He was treated in the ED with 100 mcg of levothyroxine alone and improved clinically. There were small improvements in the patient’s T4 and TSH status throughout the hospital stay. As per endocrinology, triiodothyronine would be indicated only if the patient’s status deteriorated or failed to improve on a maximum daily dose of T4. Our patient had one episode of bradycardia and hypothermia, and at this time levothyroxine was increased to 100 mcg daily and his vitals and mental status improved back to baseline. 

Despite the reported efficacy of triiodothyronine at low doses in conjunction with levothyroxine, triiodothyronine was not indicated in our patient. The patient was stable and continued to improve clinically and objectively with levothyroxine alone. These findings propose a more conservative yet effective approach to the treatment of myxedema coma. The dose maximization of levothyroxine should be first line for a patient with suboptimal improvement in condition. Meticulous clinical observation of the patient’s status is critical in order to recognize the efficacy of treatment. If the patient still fails to improve, triiodothyronine should be added at a low dose. This approach is especially integral to the treatment of elderly patients and those with underlying cardiac or respiratory conditions. We believe the judicious use of triiodothyronine can avoid catastrophic outcomes and improve the mortality rate of myxedema coma [5-7].

## Conclusions

Although myxedema coma is now a rare occurrence given the widespread availability of TSH assay and frequent monitoring by primary care physicians, it is a life-threatening event and emergency treatment is warranted. We request physicians to have this differential in mind in appropriate clinical scenarios. Also, we encourage our readers to perform more studies on treatment with thyroxine alone versus the combination of triiodothyronine and thyroxine.
